# Coarse-Grained Simulations Using a Multipolar Force Field Model

**DOI:** 10.3390/ma11081328

**Published:** 2018-07-31

**Authors:** Shuo-Feng Chiu, Sheng D. Chao

**Affiliations:** Institute of Applied Mechanics, National Taiwan University, Taipei 10617, Taiwan

**Keywords:** coarse-grained force field, multipolar expansion, intermolecular potential, fullerene

## Abstract

This paper presents a coarse-grained molecular simulation for fullerenes based on a multipolar expansion method developed previously. The method is enabled by the construction of transferable united atoms potentials that approximate the full atomistic intermolecular interactions, as obtained from ab initio electronic structure calculations supplemented by empirical force fields and experimental data, or any combination of the above. The resultant series contains controllable moment tensors that allow to estimate the errors, and approaches the all-atom intermolecular potential as the expansion order increases. We can compute the united atoms potentials very efficiently with a few interaction moment tensors, in order to implement a parallel algorithm on molecular interactions. Our simulations describe the mechanism for the condensation of fullerenes, and they produce excellent agreement with benchmark fully atomistic molecular dynamics simulations.

## 1. Introduction

Molecular dynamics (MD) simulations have become an indispensable tool for understanding the processes of microscopic structures, dynamics, and thermodynamics occurring on chemically and/or biologically interesting length/time scales [1,2,3]. It provides a direct exploration of atomistic details, and is well suited for the study of relatively complex systems. Based on the Schrödinger equation, the molecular orbital theory, the density functional theory and semi-empirical methods have been usually used to calculate the electronic wave functions of atoms and molecules in quantum chemical calculations, thus obtaining more quantitative information on various molecular and materials systems. However, it is not straightforward to efficiently model these systems because the mesoscopic scales of organizations associated with many large systems, such as soft matters, bio-polymers, or big nanostructures, are limited to the length/time scales of micrometers/nanoseconds. These structures are too large for MD simulations and too complex to be described by simple analytical models. An alternative method for extending these scales, called the coarse-grained (CG) model, provides a simple rescaling of the intermolecular effective interactions with a reduced number of degrees of freedom [4,5,6]. Balancing the reduction of variables while keeping the essential properties of polymer chains retained is the essence of CG methods.

In past years, there has been a growing interest in the development and integration of workable multiscale simulation schemes [7,8,9,10,11,12]. One of these approaches is based on the reduced representation of molecular structures. This scheme often contains the following steps: (1) Grouping atoms together and treating them as fewer interaction segments, and (2) constructing an effective force field based on a CG procedure for the interaction segments. The interactions among segments are usually described by some appropriate potential energy functions. These are almost invariably represented by a parameterized pre-selected analytical form of the CG potential, such as the Lennard-Jones potential. The interactions among segments are either reduced to steric interactions that prevent occupation of the same lattice points by two or more polymer segments, or simple analytical effective potentials that are used to determine segment-segment interactions. The connectivity of the polymer chains is maintained by ensuring that consecutive polymer segments in the same chain share a node at all times. The multiscale coarse-graining (MS-CG) is another method of reduction of structure proposed by Izvekov and Voth (2005) [13]. The foundation of this approach is the force-matching method used to develop effective empirical force fields from ab initio MD data. It should yield the equilibrium distribution of the CG coordinates identical to the underlying atomistic model. The procedure starts from an initial guess form for the CG potential from a reference distribution. These are principally based on simple potential functions like pairwise additive two-body potentials [14], three-body potentials [15] and many-body potentials [16].

In previous studies, we have simulated thermodynamical properties successfully by using first-principles density functional calculations and ab initio methods to develop the intermolecular potential energy surfaces (PES), such as hydrocarbons interaction potentials. We evaluate the performance of the simulation results by directly comparing ab initio molecular dynamics simulations with experiments [17,18,19]. In this paper, we develop a systematic approach for the coarse-grained rigid blob (CGRB) models, which can provide a hierarchy of multiscale numerical tests. In particular, this model considers both microscopic and mesoscopic characteristics of a studied system. The main obstacle resides at the technical difficulty of incorporating both computational efficiency and microscopic details at the same time. To solve this problem, we derive a multipolar series expansion, which is more efficient than the conventional multipole expansion expressed either with the Cartesian or the spherical tensor formalism [20,21]. As the most important utility for the CGRB model, we develop a systematic means to construct coarse-grained intermolecular interactions for molecular dynamics simulations for soft matter systems. This is a self-consistent working scheme for mapping constitutive finer structures and atomic interactions to coarser but accurate intermolecular interactions.

## 2. Materials and Methods

The approach presented in this work employs classical mechanics to describe the dynamical behavior of a collection of anisotropic united atoms retaining atomic-scale properties. To improve the description of spatially-varying shapes of such united atoms, we developed a multipolar force field model based on either their types or the relative orientations with respect to the centers of mass axes. The series can analytically sum up the contributions term-by-term to the effective inter-blob interaction. After the specification of the positions for the CG model, we performing a reduced analysis of the intermolecular potential energy functions of the individual blobs. Once the motion behavior has been described, the most significant task is to build the potential energy for the united atoms from which the forces and torques could be derived. 

### 2.1. Interblob Interaction Energy Model 

**Figure 1:**
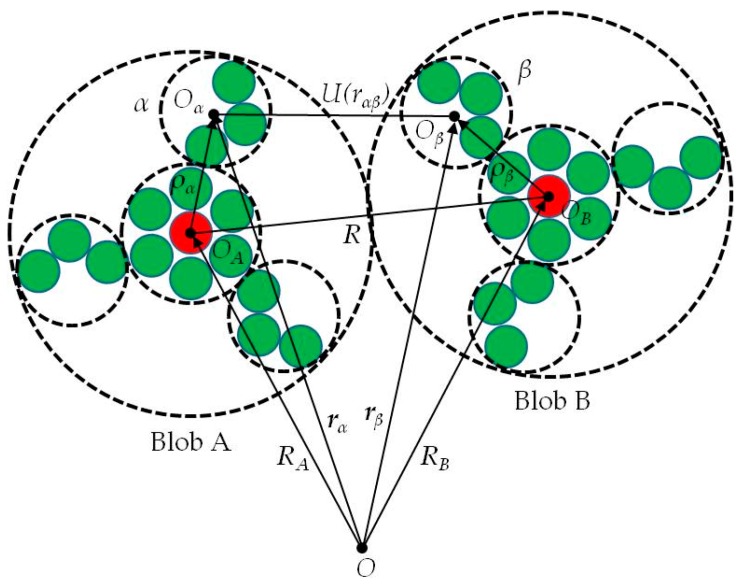
Schematic illustration of two blobs in a laboratory fixed coordinate system. Atoms are represented by small solid circles.

In this section, the working equations for governing the interblob interaction energy are derived. Consider two rigid blobs, *A* and *B*, interacting with each other, as shown in Figure 1. Suppose we can generate individual united atoms (α, β,…) for each constituent functional group, where the position of center of mass *O_A_* of blob A is represented by the vector ***R****_A_*. The position vector of a united atom α in blob A is denoted by ***ρ****_α_*. The distance between two united atoms is denoted by ***r****_αβ_*. The intermolecular potential energy between the united atoms *α* and *β* is $U\left(r_{\alpha \beta }\right)$. Moreover, structural assumptions, which are embodied in our models and are divided to obtain the exact energy E(R, Ω), can be evaluated as accurately and efficiently as possible. Here, the symbol Ω is the angular coordinate for determining the relative orientation of the two blobs. The first assumption is that all of the united atoms interact with each other through the same potential function U. The second assumption is that with each of these united atoms, we can associate a single position vector ***ρ****_α_* ($\alpha =1,2\ldots ,N_{A}$). For example, considering blob A, shown in Figure 1, we can assign *N_A_* = 4 interaction regions with chemically bonded groups of atoms. The third assumption is that E(R, Ω) can be approximated by an interaction energy V(R, Ω), described by the sum of pairwise energy functions between united atoms in the following Equation (1):

(1) $$E\left(R,\;\Omega \right)\approx V\left(R,\;\Omega \right)=\sum_{\alpha =1}^{N_{A}}\sum_{\beta =1}^{N_{B}}U_{AB}\left(r_{\alpha \beta }\right)\;$$

Suppose that the pair of united atoms interacts with each other via a potential function *U_AB_*(*r_αβ_*); namely

(2) $$r_{\alpha \beta }=\left|r_{\alpha }-r_{\beta }\right|=\left|\left(R_{A}+\rho_{\alpha }\right)-\left(R_{B}+\rho_{\beta }\right)\right|=\left|R+\rho_{\alpha \beta }\right|\;$$

The potential function *U_AB_(r_αβ_*) can be expressed as a Taylor series expansion about the point *r_αβ_ = R* and can be truncated to fourth-order terms. If the attention is restricted to *R > ρ_αβ_*, where the distance R=|R| and $\rho_{\alpha \beta }=\left|\rho_{\alpha \beta }\right|$, $U_{AB}\left(r_{\alpha \beta }\right)$ is given by the following equation:

(3) $$\begin{array}{ll} U_{AB}\left(r_{\alpha \beta }\right) & =\;\sum_{\left(m,n\right)}V^{\left(mn\right)}\Theta_{\alpha \beta }^{\left(mn\right)}\\ & =U\left(R\right)+\frac{\partial U}{\partial R}\left(\hat{R}\cdot \rho_{\alpha \beta }\right)+\frac{1}{2}\left(\frac{1}{R}\frac{\partial U}{\partial R}\right)\rho_{\alpha \beta }^{2}+\frac{1}{2}\left(\frac{\partial^{2}U}{\partial R^{2}}-\frac{1}{R}\frac{\partial U}{\partial R}\right){\left(\hat{R}\cdot \rho_{\alpha \beta }\right)}^{2}\\ & +\frac{1}{6}\left[\frac{\partial^{3}U}{\partial R^{3}}-\frac{3}{R}\left(\frac{\partial^{2}U}{\partial R^{2}}-\frac{1}{R}\frac{\partial U}{\partial R}\right)\right]{\left(\hat{R}\cdot \rho_{\alpha \beta }\right)}^{3}+\frac{1}{8}\left[\frac{1}{R^{2}}\left(\frac{\partial^{2}U}{\partial R^{2}}-\frac{1}{R}\frac{\partial U}{\partial R}\right)\right]\rho_{\alpha \beta }^{4}\\ & +\frac{1}{4}\left[\frac{1}{R}\left(\frac{\partial^{3}U}{\partial R^{3}}-\frac{3}{R}\left(\frac{\partial^{2}U}{\partial R^{2}}-\frac{1}{R}\frac{\partial U}{\partial R}\right)\right)\right]\rho_{\alpha \beta }^{2}{\left(\hat{R}\cdot \rho_{\alpha \beta }\right)}^{2}\\ & +\frac{1}{24}\left[\frac{\partial^{4}U}{\partial R^{4}}-\frac{6}{R}\frac{\partial^{3}U}{\partial R^{3}}+\frac{15}{R^{2}}\left(\frac{\partial^{2}U}{\partial R^{2}}-\frac{1}{R}\frac{\partial U}{\partial R}\right)\right]{\left(\hat{R}\cdot \rho_{\alpha \beta }\right)}^{4}+\cdots , \end{array}\;$$

where:

(4) $$V^{\left(mn\right)}=\frac{1}{n!\left(m-n\right)!!}R^{n}{\left(\frac{1}{R}\frac{\partial }{\partial R}\right)}^{\left(m+n\right)/2}U\left(R\right)\;$$

with m!!=m(m−2)(m−4)⋯(4)(2) and:

(5) $$\Theta_{\alpha \beta }^{\left(mn\right)}={\left(\hat{R}\cdot \rho_{\alpha \beta }\right)}^{n}\rho_{\alpha \beta }^{m-n}\;$$

where $\hat{R}$ is the unit vector along the R direction. Note that the integer pair (*m*, *n*) of values separated by a comma are the indices of an element. The values follow the form (0, 0), (2, 0), (2, 2), (4, 0), (4, 2), (4, 4), … for even parts and (1, 1), (3, 1), (3, 3), … for odd parts. Therefore, we can obtain the constraint for the integer pair (*m*, *n*):

(6) $$n=\left\{ \begin{array}{l} m,m-2,m-4,\ldots ,0\;m\;\mathrm{even},\\ m,m-2,m-4,\ldots ,1\;m\;\mathrm{odd}. \end{array}\right.\;$$

where the sums of m+n and m−n are always even. Thus, each term of the potential energy (Equation (3)) can be ensured to be a scalar by these indices. 

By substituting Equation (3) into Equation (1), the interblob potential can therefore be expressed by the following Equations (7) and (8):

(7) $$V\left(R,\Omega \right)=\sum_{\left(m,m\right)}V^{\left(mn\right)}\Theta^{\left(mn\right)}\;$$

(8) $$\Theta^{\left(mn\right)}=\sum_{\alpha \beta }\Theta_{\alpha \beta }^{\left(mn\right)}\;$$

In this methodology, the potential energy is thus calculated as the sum of products of the radial parts, $V^{\left(mn\right)}$, and the angular parts, $\Theta^{\left(mn\right)}$, of the system. Obviously, it is feasible to reduce the degree of freedom of a fully atomic molecular dynamics simulation of a complex system by including only those features that are necessary to characterize the system details. With this approach, the tensor characteristics play a very important role in the possibility of manipulation of the parameters for molecule-specific interactions. The resulting angular parts are given by, (see Appendix A):

(9) $$\begin{array}{ll} \sum_{\alpha \beta }\Theta_{\alpha \beta }^{\left(mn\right)}= & \sum_{k=0}^{n}\sum_{l=0}^{m-n}{\left(N_{A}\right)|}_{n-k,\;m-n-l=0}{\left(N_{B}\right)|}_{k,\;l=0}{\left(-1\right)}^{\left(k+l\right)}\cdot \\ & \left[\left(\begin{array}{c} n\\ k \end{array}\right)Tr^{\left(m-n-k\right)}{\hat{R}}^{\left(n-k\right)}{\langle \cdot \rangle }_{\left(n-k\right)}\Gamma_{A}^{\left(n-k\right)}\Gamma_{B}^{\left(k\right)}{\langle \cdot \rangle }_{\left(k\right)}{\hat{R}}^{\left(k\right)}\right]\left[\left(\begin{array}{c} m-n\\ l \end{array}\right)Tr^{\left(m-n-l\right)}\Gamma_{A}^{\left(m-n-l\right)}Tr^{\left(l\right)}\Gamma_{B}^{\left(l\right)}\right] \end{array}\;$$

Here:

(10) $$\left(\begin{array}{c} n\\ k \end{array}\right)=\frac{n!}{k!\left(n-k\right)!}\;\mathrm{for}\;0\leq k\leq n,\;\forall k\in \mathbb{N}\;$$

(11) $$\left(\begin{array}{c} m-n\\ l \end{array}\right)=\frac{\left(m-n\right)!}{l!\left(m-n-l\right)!}\;\mathrm{for}\;0\leq l\leq m-n,\;\forall l\in \mathbb{N}\;$$

where the tensors $\Gamma_{A}^{\left(n-k\right)}$ are the (*n* − *k*) rank tensors for the blob A called the “interaction moment tensor”, and are given by:

(12) $$\Gamma_{A}^{\left(0\right)}=N_{A}\;$$

(13) $$\Gamma_{A}^{\left(1\right)}=\sum_{\alpha =1}^{N_{A}}\rho_{A\alpha }\;$$

(14) $$\Gamma_{A}^{\left(2\right)}=\sum_{\alpha =1}^{N_{A}}\rho_{A\alpha }\rho_{A\alpha }\;$$

(15) $$\Gamma_{A}^{\left(3\right)}=\sum_{\alpha =1}^{N_{A}}\rho_{A\alpha }\rho_{A\alpha }\rho_{A\alpha }\;$$

(16) $$\Gamma_{A}^{\left(4\right)}=\sum_{\alpha =1}^{N_{A}}\rho_{A\alpha }\rho_{A\alpha }\rho_{A\alpha }\rho_{A\alpha }\;$$

For example, if *m* = 2, we have:

(17) $$\Gamma_{A;kk^{\prime }}^{\left(2\right)}=\sum_{\alpha =1}^{N_{A}}\rho_{A\alpha ;k}\rho_{A\alpha ;k^{\prime }}\;$$

which is the $kk^{\prime }$ component of the rank 2 tensor. It formally contains both the shape and size properties of the blob A, and thus serves as an excellent case study in a standard multipole expansion. On the other hand, a concise symbol can be defined simply as a binary operator between two tensors.

(18) $${\langle \cdot \rangle }_{x}=\sum_{k_{1}=1}^{3}\sum_{k_{2}=1}^{3}\sum_{k_{3}=1}^{3}\cdots \sum_{k_{x}=1}^{3}\cdot ,$$

For example, when *x* = 1, it is a dot product between two tensors. The definitions of the trace components are given as follow:

(19) $$Tr^{\left(2\right)}\left(\Gamma_{A}^{\left(2\right)}\right)=\sum \rho_{A\alpha }^{2}\;$$

(20) $$Tr^{\left(2\right)}\left(\Gamma_{A}^{\left(3\right)}\right)=\sum \rho_{A\alpha }^{2}\rho_{A\alpha }\;$$

(21) $$Tr^{\left(2\right)}\left(\Gamma_{A}^{\left(4\right)}\right)=\sum \rho_{A\alpha }^{2}\rho_{A\alpha }\rho_{A\alpha }\;$$

(22) $$Tr^{\left(4\right)}\left(\Gamma_{A}^{\left(4\right)}\right)=\sum \rho_{A\alpha }^{4}\;$$

The series expansion is given in terms of molecule-specific interaction moment tensors, thus avoiding the direct calculation of all the many-body atom-atom interactions. This provides a very efficient way to handle the large and highly coupled degrees of freedom of complex systems such as soft matters. Note that although these formulas indicate that both the interaction moment tensors and the electrostatic multipole terms have a common structure in some aspects, the interpretations of the physical and symmetric properties are quite different [21]. The derivations for the inter-blob forces and torques follow similar procedures as with deriving the inter-blob potentials [20].

### 2.2. Interaction Moment Tensors

For preciseness, take the case of the potential U(R) as a Lennard-Jones (LJ) type potential:

(23) $$U\left(R\right)=4\varepsilon \left[{\left(\frac{\sigma }{R}\right)}^{\alpha }-{\left(\frac{\sigma }{R}\right)}^{\beta }\right]\;$$

where *ε* is the depth of the potential well and *σ* is the finite distance at which the intermolecular potential is zero. By substituting the Equation (23) into Equation (4), the radial parts of the potential become:

(24) $$V^{\left(mn\right)}=\frac{4\varepsilon {\left(-1\right)}^{\left(m+n\right)/2}}{n!\left(m-n\right)!!R^{m}}\left[\frac{\left(\alpha +m+n-2\right)!!}{\left(\alpha -2\right)!!}{\left(\frac{\sigma }{R}\right)}^{\alpha }-\frac{\left(\beta +m+n-2\right)!!}{\left(\beta -2\right)!!}{\left(\frac{\sigma }{R}\right)}^{\beta }\right]\;$$

**Figure 2:**
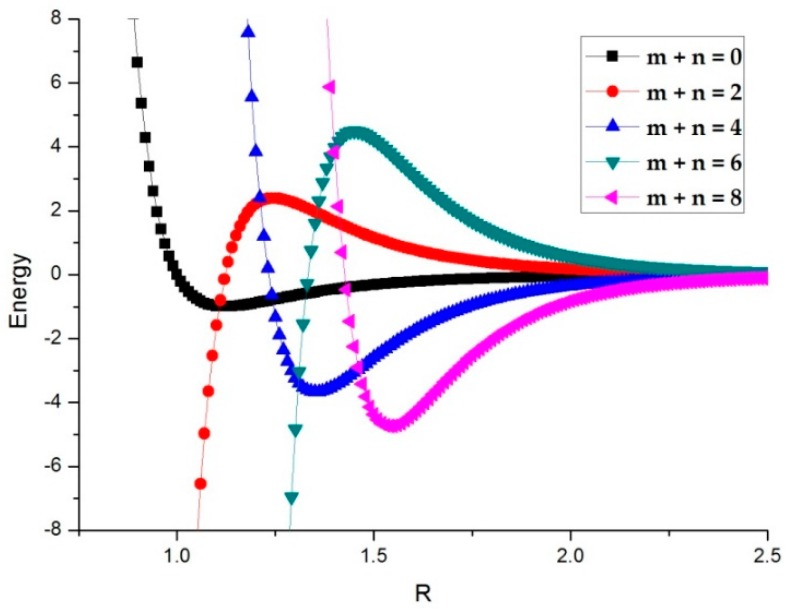
The radial part potential energy in different orders (factor m from 0 to 4). In this case, we use two standard Lennard-Jones (LJ) potential parameters, σ=ε=1, α=12 and β=6.

Figure 2 shows that the curves of the radial components move toward longer radial distances and deeper energy as the expansion order is increases.

On the other hand, as we can see from the form of angular parts of Equations (12)–(16), in general, a rank-*m* tensor with both columns and rows of sizes up to *N* can be represented by *N^m^* numbers. One should make sure that how much memory layout for storing the data is required when dealing with the multidimensional arrays. In particular, a computer programming data structure that is inherently linear enables mapping of multidimensional data to a one-dimension array. Consider a rank-*m* tensor $\Gamma^{\left(m\right)}$ in *N* dimensional space; we compute the memory location of an element from its indices by using the row-major mapping function as follows:

(25) $$\begin{array}{ll} I\left(i_{1},i_{2},\cdots ,i_{m};N\right) & =\sum_{\lambda_{1}=1}^{i_{1}-1}\frac{1}{\left(m-1\right)!}\left(N+1-\lambda_{1}\right)\left(N+2-\lambda_{1}\right)\cdots \left(N+m-1-\lambda_{1}\right)\\ & \;+\sum_{\lambda_{2}=i_{1}}^{i_{2}-1}\frac{1}{\left(m-2\right)!}\left(N+1-\lambda_{2}\right)\left(N+2-\lambda_{2}\right)\cdots \left(N+m-2-\lambda_{2}\right)\\ & \;+\cdots \\ & \;+\sum_{\lambda_{k}=i_{k-1}}^{i_{k}-1}\frac{1}{\left(m-k\right)!}\left(N+1-\lambda_{k}\right)\left(N+2-\lambda_{k}\right)\cdots \left(N+m-2-\lambda_{k}\right)\\ & \;+\cdots \\ & \;+\sum_{\lambda_{m-1}=i_{m-2}}^{i_{m-1}-1}\frac{1}{1!}\left(N+1-\lambda_{m-1}\right)\\ & \;+\sum_{\lambda_{m}=i_{m-1}}^{i_{m}}\frac{1}{0!} \end{array}\;$$

where *I* is the location of index number for the one dimension array, $i_{m}$ is the index number of a rank-*m* tensor, and λ is the index operator from 0 to *m*. If we make the symmetric constraint for the tensors, we have:

(26) $$\begin{array}{l} \sum_{\lambda =1}^{N-1}\frac{1}{\left(m-1\right)!}\left(N+1-\lambda \right)\left(N+2-\lambda \right)\cdots \left(N+m-1-\lambda \right)+1\\ =\left(\begin{array}{c} N+m-1\\ m \end{array}\right)=\left(\begin{array}{c} N+m-1\\ N-1 \end{array}\right) \end{array}\;$$

Thus, the interaction moment tensor can be retrieved by the index mapping function. Table 1 shows the memory layout of a rank-4 tensor array with *N* = 3 in row-major format.

## 3. Results and Discussion

Applications of a fully atomic MD simulation were still limited by the length and time scales despite the rapid increase of computing power. A well-designed CG model opens up the possibility for long simulation processes with an efficiency over that of the fully atomic MD. Here, we consider a system of fullerene molecules. For a fullerene, 60 carbon atoms are arranged at the vertexes of a truncated icosahedron which could roughly fit into a sphere of about 7.1 Å in diameter. In this section, we use both the all-atom and the CG methods to study the C60–C60 interactions and provide application-based benchmarks using the neutral C60 dimer as a test case.

### 3.1. Structure of C60

Calculating the bonding structure of the fullerene dimer was the first step toward the molecular dynamics simulation. The structure of fullerene was optimized at the ωB97XD/6-31G(d) level of theory. The density functional theory (DFT) calculations were carried out using a Gaussian 09 program package [22]. In the present work, the optimized structure was found and the molecular point group of C60 corresponded to *Ih* symmetry. Moreover, for comparison, single bonds (C–C) and double bonds (C=C) of the dimer as well as the monomer showed clearly that the bond length was not affected by process of dimerization, as shown in Table 2.

### 3.2. Potential Curve Fitting

**Figure 3:**
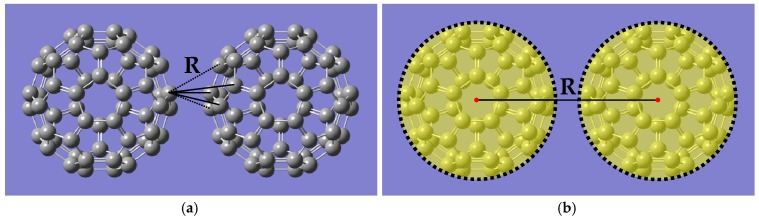
Discrete summation for the calculation of the intermolecular interaction potential between two approaching fullerenes. (**a**) All-atom model: 3600 times per step, and (**b**) coarse-grained model: one time per step.

In the all-atom process, the value of energy was calculated by summing up all interactions between carbon atoms on any two fullerene molecules. In the coarse-grained process, the information of these carbon atoms interactions were mapped onto one single point to the center of mass of the molecular structure as shown in Figure 3. The pairwise atomic carbon-carbon potential were modeled by the Morse potential, as shown in Equation (27):

(27) $$U(R)=D_{e}\left[e^{-2\alpha \left(R-R_{0}\right)}-2e^{-\alpha \left(R-R_{0}\right)}\right]\;$$

where *D_e_* is the well depth that corresponds to the dissociation energy, *α* is a parameter controlling the width of the potential well, and *R*_0_ is the equilibrium bond distance. The interaction potential was obtained by fitting a Morse potential function to empirical force field data. Each fullerene molecule was treated as a collection of separate united atoms by using interaction moment tensors. We performed molecular dynamics calculations in which the potential formula was truncated up to the fourth order. In order to obtain the forms of the angular parts of the potential, the independent components of interaction moment tensors were fitted as shown in Table 3.

**Figure 4:**
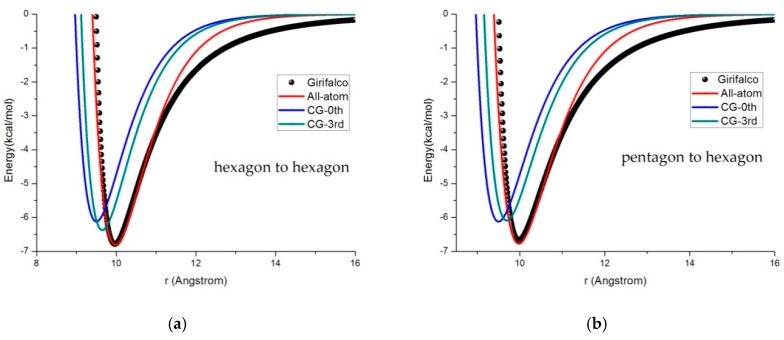

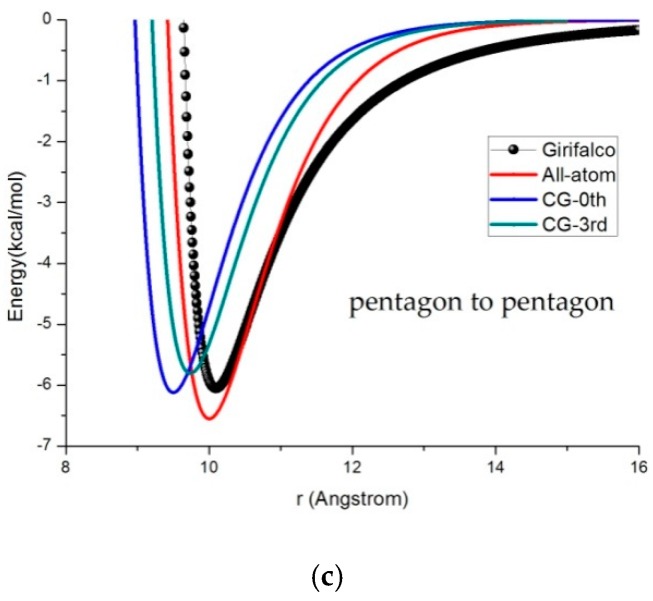
Comparison of the fitting curves (Girifalco potential, Morse potential with the all-atom model, 0th CG, and the 3rd CG model). (**a**) Hexagonal face-to-hexagonal face, (**b**) hexagonal face-to-pentagonal face and (**c**) pentagonal face-to-pentagonal face configurations.

We noted that the potential energy expansion of the CG model was truncated after the third-order because of the dominant effects due to the symmetrical properties of the C60 molecules. The fitting parameters of the Morse potential to the Girifalco potential [24] that we obtained are *R*_0_ = 4.1 Å, *D_e_* = 0.074 kcal/mol and *α* = 1.3 for the all-atom work. In the CG work, we fitted the potential curves using both the zeroth-order for *R*_0_ = 9.5 Å, *D_e_* = 0.0017 kcal/mol, and *α* = 1.3, and the third-order for *R*_0_ = 9.65 Å, *D_e_* = 0.00177 kcal/mol, and *α* = 1.3. For both the all-atom and the CG models, the potential energy curves that we calculated on a pair of fullerene molecules at pentagonal face-to-pentagonal face, hexagonal face-to-pentagonal face, and hexagonal face-to-hexagonal face configurations, respectively, are shown in Figure 4.

### 3.3. Radial Distribution Function

**Figure 5:**
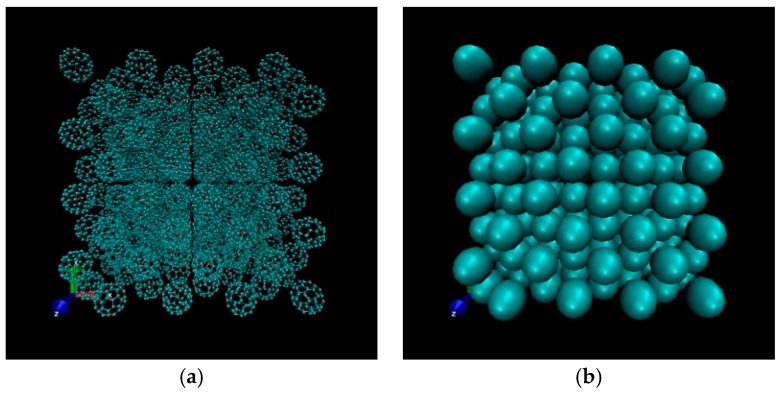
Initial state of C60 molecules in a molecular dynamics (MD) simulation. (**a**) All-atom model. (**b**) CG model.

**Figure 6:**
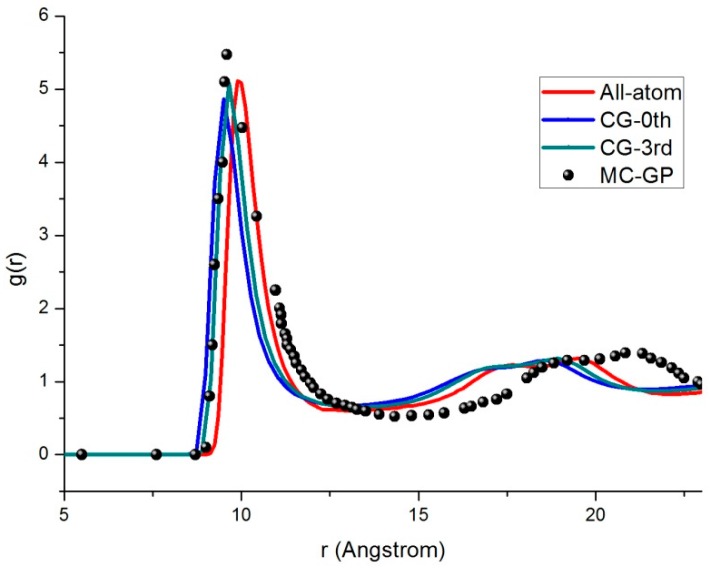
Radial distribution functions of the center of mass for different models representing fullerene molecules.

To further ensure the validity of the computational techniques developed here, we compared CG models with all-atom molecular dynamics. In the initial state, 256 molecules were arranged in an face-centered cubic structure with the three-dimensional periodic boundary condition (PBC) as a model of liquid-phase molecules, as shown in Figure 5. The equation of motion was integrated with a leapfrog Verlet integration algorithm, and the cut-off radius was set to 3σ* (where σ* is the MD length unit). Newton’s equations of motion for the center of mass motions and Euler equations for the rotational motions were integrated. Our programs for molecular dynamics simulation were carried out in canonical ensemble (NVT) using the home-modified codes provided by reference [25]. We analyzed from MD simulation data several relevant observables to characterize the thermodynamics properties of C60 molecule in the liquid state. Figure 6 presents the simulated results using the different models for the radial distribution function (RDF) of fullerene at temperature *T* = 1529 K and density *ρ* = 1.219 g/cm^3^. Overall, the RDF curves displayed a typical behavior in molecular dynamics with an asymptotic value of 1, which represented the probability of finding the center of a molecule with a given distance. The result showed that the RDFs of the C60 molecules had sharp first peaks for both all-atom and CG models, which indicated close interactions among the two fullerenes. In the case of the all-atom model, g(r) had its maximal value of 5.11 located at a distance of 9.89 Å. On the other hand, in the case of both the zeroth-order CG and the third-order CG models, the first peak values were 4.87 and 5.09 of g(r) and the distances are located at the values of 9.5 Å and 9.65 Å, respectively. It was seen that when the expansion was up to the third-order, the present model performed equally well in the first peak region as an all-atom model. We also compared the RDF obtained from Fernandes et al. [26] using the Monte Carlo method in Figure 6. We see that our CG model reproduced the full atomistic simulation results. 

In principle, the precision of the CG model could be improved by adding higher-order terms. However, the number of tensor parameters increases dramatically and we had to truncate the series at a suitable order. For fullerenes, the observed differences between the CG model and the all-atom simulations were due to such truncations.

**Figure 7:**
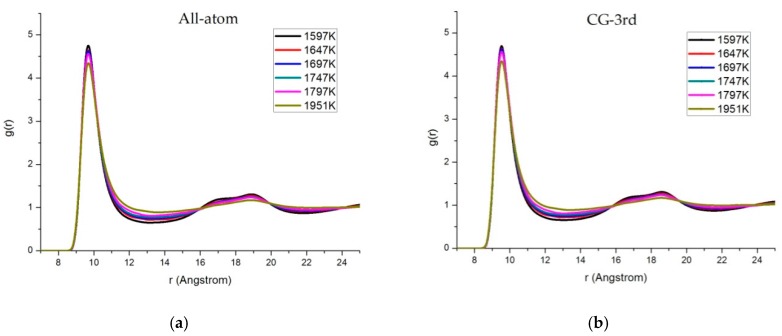
Temperature effect of RDFs for (**a**) the all-atom model and (**b**) the third-order CG model.

Figure 7 presents the temperature effect of RDFs for C60 obtained by the all-atom and the CG-3rd models. We employed thermostats by rescaling the velocity along the steam line. The temperature was changed in intervals of 50 K from 1597 K to 1797 K and the critical point 1951 K [26], respectively. In particular above 1951 K, since the RDF did not have distinct peak structures except for the first peak; the line became smooth after 13 Å in the high temperature region. The first peak of the RDF tended to decrease as the temperature increased, which indicated a relatively weak interaction force when the system was approaching the critical point.

### 3.4. Velocity Autocorrelation Function

**Figure 8:**
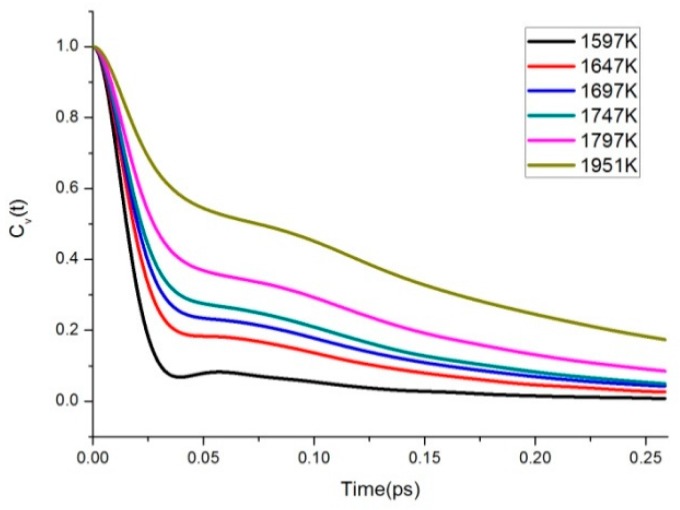
The VAFs of C60 molecule at several temperature conditions.

Atomic velocity autocorrelation function (VAF), a key quantity in the microscopic dynamics of a MD simulation, usually provides kinetic and thermodynamic information in the time evolution [27]. The expression for calculating VAF is:

(28) $$C_{v}\left(t\right)=\langle v\left(t\right)v\left(0\right)\rangle /\langle v^{2}\left(0\right)\rangle \;$$

where v is the translational velocity of the center of mass of molecule. We showed the VAFs for several phase conditions considered in this paper in Figure 8. Overall the VAFs decay rapidly in the range of 0 ps to 0.1 ps and become nearly uncorrelated after 0.2 ps. It can be seen clearly that the curve of *C_v_*(*t*) changed to a deeper dip with lower temperature, which is a typical behavior of the liquid state. However, as the temperature increased, the VAFs progressively became smooth and decayed more slowly to zero.

For high temperatures, the long-time tails of the VAF decayed slowly. To improve the precision, we run some longer-time simulations with double system size. For the longest time span (up to 0.5 ps), the tail part contribution to the VAF is roughly 10% on average. This can be largely reduced by scaling the system size.

### 3.5. Self-Diffusion Coefficient

We also calculated the self-diffusion coefficients as a function of temperature. The diffusion coefficient *D* is obtained by the Green–Kubo formula [28,29]:

(29) $$D=\frac{1}{3N}\int_{0}^{\infty }\langle \sum_{i}^{N}v_{i}\left(t\right)v_{i}\left(0\right)\rangle dt\;$$

**Figure 9:**
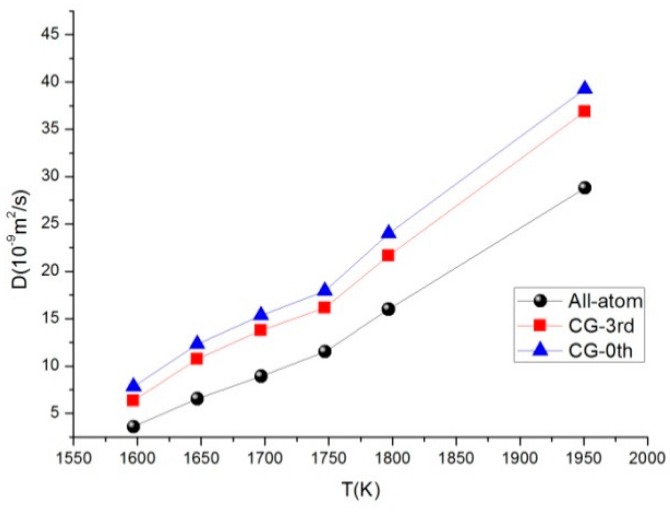
The temperature dependence of the diffusion coefficient of C60 in different models.

We performed numerical integration of the VAFs data and showed the temperature effects with all-atom models for comparison. Figure 9 shows that self-diffusion coefficient of C60 increased significantly with increasing temperature. In Table 4, we present the comparison of the calculated self-diffusion coefficients for details in this work. The result shows that the curves of *D* in CG model overestimated the all-atom model, but the overall trends were similar. 

## 4. Conclusions

In this paper we construct a CGRB model based on multipolar expansion force field implementation. It offers an appropriate potential formula for describing the intermolecular interaction patterns and is suitable for parallel architecture. Moreover, it provides a computationally efficient and controllable way to approximate the all-atom simulation, thus avoiding complex intramolecular dynamics calculations. In molecular simulations, our fitting curves show good approximations of this multipolar expansion method to the all-atom model. We also calculate the RDFs, VAFs, and diffusion constants characterizing the thermodynamic properties of the C60 molecule in the liquid state, using both the all-atom and the CG methods. The results show this CGRB model approach successfully reproduces the thermodynamic properties of the all-atom model. This flexibility of the CGRB model provides possibilities for performing a hierarchy of multiscale simulations.

Because our CG models are based on rigid blob assumption, the internal entropy from the inner degrees of freedom is neglected. For fullerenes, the entropic contribution to the free energy is estimated to be about only 0.2%. However, for more flexible polymers, those effects can be significant, such as those in rubber elasticity. This effect is currently under study in our group, which can provide extensions of the CGRB model [30,31].

## Figures and Tables

**Table 1:** List of the angular components and their memory locations. Row indices go over rows from top to bottom; column indices go over columns from left to right for a matrix.

Rank-m Tensors	Independent Components	Memory Location
$\Gamma_{0}^{\left(0\right)}$	N	$\Gamma_{0}$
$\Gamma_{i_{1}}^{\left(1\right)}$	$\Gamma_{1}^{\left(1\right)}$, $\Gamma_{2}^{\left(1\right)}$, $\Gamma_{3}^{\left(1\right)}$	$\Gamma_{1}$, $\Gamma_{2}$, $\Gamma_{3}$
$\Gamma_{i_{1},i_{2}}^{\left(2\right)}$	$\Gamma_{11}^{\left(2\right)}$, $\Gamma_{12}^{\left(2\right)}$, $\Gamma_{13}^{\left(2\right)}$, $\Gamma_{21}^{\left(2\right)}$, $\Gamma_{22}^{\left(2\right)}$, $\Gamma_{23}^{\left(2\right)}$	$\Gamma_{4}$, $\Gamma_{5}$, $\Gamma_{6}$, $\Gamma_{7}$, $\Gamma_{8}$, $\Gamma_{9}$
$\Gamma_{i_{1},i_{2},i_{3}}^{\left(3\right)}$	$\Gamma_{111}^{\left(3\right)}$, $\Gamma_{112}^{\left(3\right)}$, $\Gamma_{113}^{\left(3\right)}$, $\Gamma_{122}^{\left(3\right)}$, $\Gamma_{123}^{\left(3\right)}$, $\Gamma_{133}^{\left(3\right)}$, $\Gamma_{222}^{\left(3\right)}$, $\Gamma_{223}^{\left(3\right)}$, $\Gamma_{233}^{\left(3\right)}$, $\Gamma_{333}^{\left(3\right)}$	$\Gamma_{10}$, $\Gamma_{11}$, $\Gamma_{12}$, $\Gamma_{13}$, $\Gamma_{14}$, $\Gamma_{15}$, $\Gamma_{16}$, $\Gamma_{17}$, $\Gamma_{18}$, $\Gamma_{19}$
$\Gamma_{i_{1},i_{2},i_{3},i_{4}}^{\left(4\right)}$	$\Gamma_{1111}^{\left(4\right)}$, $\Gamma_{1112}^{\left(4\right)}$, $\Gamma_{1113}^{\left(4\right)}$, $\Gamma_{1122}^{\left(4\right)}$, $\Gamma_{1123}^{\left(4\right)}$, $\Gamma_{1133}^{\left(4\right)}$, $\Gamma_{1222}^{\left(4\right)}$, $\Gamma_{1223}^{\left(4\right)}$, $\Gamma_{1233}^{\left(4\right)}$, $\Gamma_{1333}^{\left(4\right)}$, $\Gamma_{2222}^{\left(4\right)}$, $\Gamma_{2223}^{\left(4\right)}$, $\Gamma_{2233}^{\left(4\right)}$, $\Gamma_{2333}^{\left(4\right)}$, $\Gamma_{3333}^{\left(4\right)}$	$\Gamma_{20}$, $\Gamma_{21}$, $\Gamma_{22}$, $\Gamma_{23}$, $\Gamma_{24}$, $\Gamma_{25}$, $\Gamma_{26}$, $\Gamma_{27}$, $\Gamma_{28}$, $\Gamma_{29}$, $\Gamma_{30}$, $\Gamma_{31}$, $\Gamma_{32}$, $\Gamma_{33}$, $\Gamma_{34}$

**Table 2:** Several density functional theory (DFT) methods have been assessed by evaluating the optimized structural parameters of the C60 molecule.

Method	C–C (Å)	C=C (Å)	r (Å)
Experiment [23]	1.455	1.391	3.545
RHF/STO-3G	1.453	1.367	3.524
HF/6-31G(d, p)	1.449	1.373	3.523
B3LYP/6-31G(d, p)	1.453	1.395	3.550
ωB97XD/6-31G(d)—monomer	1.450	1.386	3.535
ωB97XD/6-31G(d)—dimer	1.452	1.388	3.538

**Table 3:** Fitting parameters for the interaction moment tensor.

Rank-m Number	Memory Location	Fitting Parameters
0	$\Gamma_{0}$	60
1	$\Gamma_{1}$, $\Gamma_{2}$, $\Gamma_{3}$	0.00000, 0.00002, 0.00002
2	$\Gamma_{4}$, $\Gamma_{5}$, $\Gamma_{6}$,	251.57483, 0.00871, 0.02755,
	$\Gamma_{7}$, $\Gamma_{8}$, $\Gamma_{9}$	251.57513, −0.03941, 251.6387
3	$\Gamma_{10}$, $\Gamma_{11}$, $\Gamma_{12}$,	0.00190, 0.00288, −0.00735,
	$\Gamma_{13}$, $\Gamma_{14}$,$\Gamma_{15}$,	−0.00382, −0.00095, 0.00172,
	$\Gamma_{16}$, $\Gamma_{17}$, $\Gamma_{18}$, $\Gamma_{19}$	0.01110, −0.00650, −0.01328, 0.01462
4	$\Gamma_{20}$, $\Gamma_{21}$, $\Gamma_{22}$,	1898.65174, −0.08173, 0.23940,
	$\Gamma_{23}$, $\Gamma_{24}$, $\Gamma_{25}$,	632.88311, −0.12178, 633.07361,
	$\Gamma_{26}$, $\Gamma_{27}$, $\Gamma_{28}$,	−0.07104, 0.07556, 0.02058,
	$\Gamma_{29}$, $\Gamma_{30}$, $\Gamma_{31}$,	0.23205, 1898.67454, −0.34235,
	$\Gamma_{32}$, $\Gamma_{33}$, $\Gamma_{34}$	633.05680, −0.31836, 1899.74600

**Table 4:** The self-diffusion coefficients using the Green–Kubo formula as compared to all models.

T (K)	ρ (g/cm^3^)	D (10^−9^ m^2^/s)		
		All-Atom Model	CG-3rd Model	CG-0th Model
1597	1.2195	3.598	6.330	7.818
1647	1.0163	6.544	10.746	12.327
1697	0.9326	8.915	13.760	15.360
1747	0.8728	11.514	16.163	17.914
1797	0.7353	16.001	21.672	23.983
1951	0.5058	28.797	36.882	39.239

## Appendix A

Additional information: Angular parts, $\Theta^{\left(mn\right)}$, (*m*, *n*) run over pairs (0, 0), (1, 1), (2, 0), (2, 2), (3, 1), (3, 3), (4, 0), (4, 2), (4, 4).

(A1) $$\Theta^{\left(00\right)}=N_{A}N_{B}\;$$

(A2) $$\Theta^{\left(11\right)}=N_{B}\hat{R}\cdot \Gamma_{A}^{\left(1\right)}-N_{A}\hat{R}\cdot \Gamma_{B}^{\left(1\right)}\;$$

(A3) $$\Theta^{\left(20\right)}=N_{B}Tr^{\left(2\right)}(\Gamma_{A}^{\left(2\right)})-2\Gamma_{A}^{\left(1\right)}\Gamma_{B}^{\left(1\right)}+N_{A}Tr^{\left(2\right)}(\Gamma_{B}^{\left(2\right)})\;$$

(A4) $$\Theta^{\left(22\right)}=N_{B}{\hat{R}}^{2}{\langle \cdot \rangle }_{2}\Gamma_{A}^{\left(2\right)}-2\hat{R}\cdot \Gamma_{A}^{\left(1\right)}\Gamma_{B}^{\left(1\right)}\cdot \hat{R}+N_{A}{\hat{R}}^{2}{\langle \cdot \rangle }_{2}\Gamma_{B}^{\left(2\right)}\;$$

(A5) $$\begin{array}{ll} \Theta^{\left(31\right)} & =N_{B}\hat{R}\cdot Tr^{\left(2\right)}\left(\Gamma_{A}^{\left(3\right)}\right)-2\hat{R}\cdot \Gamma_{A}^{\left(2\right)}\Gamma_{B}^{\left(1\right)}+\hat{R}\cdot \Gamma_{A}^{\left(1\right)}Tr^{\left(2\right)}\left(\Gamma_{B}^{\left(2\right)}\right)-Tr^{\left(2\right)}\left(\Gamma_{A}^{\left(2\right)}\right)\Gamma_{B}^{\left(1\right)}\cdot \hat{R}\\ & +2\Gamma_{A}^{\left(1\right)}\cdot \Gamma_{B}^{\left(2\right)}\cdot \hat{R}-N_{A}Tr^{\left(2\right)}\left(\Gamma_{B}^{\left(3\right)}\right)\cdot \hat{R} \end{array}\;$$

(A6) $$\Theta^{\left(33\right)}=N_{B}{\hat{R}}^{3}{\langle \cdot \rangle }_{3}\Gamma_{A}^{\left(3\right)}-3{\hat{R}}^{2}{\langle \cdot \rangle }_{2}\Gamma_{A}^{\left(2\right)}\Gamma_{B}^{\left(1\right)}\cdot \hat{R}+3\hat{R}\cdot \Gamma_{A}^{\left(1\right)}\Gamma_{B}^{\left(2\right)}{\langle \cdot \rangle }_{2}{\hat{R}}^{2}-N_{A}\Gamma_{B}^{\left(3\right)}{\hat{R}}^{3}{\langle \cdot \rangle }_{3}\;$$

(A7) $$\begin{array}{ll} \Theta^{\left(40\right)} & =N_{B}Tr^{\left(4\right)}(\Gamma_{A}^{\left(4\right)})+2Tr^{\left(2\right)}\left(\Gamma_{A}^{\left(2\right)}\right)Tr^{\left(2\right)}\left(\Gamma_{A}^{\left(2\right)}\right)+N_{A}Tr^{\left(4\right)}(\Gamma_{B}^{\left(4\right)})-4Tr^{\left(2\right)}\left(\Gamma_{A}^{\left(3\right)}\right)\cdot \Gamma_{B}^{\left(1\right)}\\ & -4\Gamma_{A}^{\left(1\right)}Tr^{\left(2\right)}\left(\Gamma_{B}^{\left(3\right)}\right)+4\Gamma_{A}^{\left(2\right)}{\langle \cdot \rangle }_{2}\Gamma_{B}^{\left(2\right)} \end{array}\;$$

(A8) $$\begin{array}{ll} \Theta^{\left(42\right)} & =N_{B}{\hat{R}}^{2}{\langle \cdot \rangle }_{2}Tr^{\left(2\right)}(\Gamma_{B}^{\left(4\right)})+{\hat{R}}^{2}{\langle \cdot \rangle }_{2}\Gamma_{A}^{\left(2\right)}Tr^{\left(2\right)}(\Gamma_{B}^{\left(2\right)})-2\hat{R}\cdot \Gamma_{A}^{\left(3\right)}\cdot \Gamma_{B}^{\left(1\right)}\cdot \hat{R}-2\hat{R}\cdot \Gamma_{A}^{\left(1\right)}Tr^{\left(2\right)}(\Gamma_{B}^{\left(3\right)})\cdot \hat{R}\\ & -2\hat{R}\cdot Tr^{\left(2\right)}(\Gamma_{A}^{\left(3\right)})\Gamma_{B}^{\left(1\right)}\cdot \hat{R}+4\hat{R}\cdot \Gamma_{A}^{\left(2\right)}\cdot \Gamma_{B}^{\left(2\right)}\cdot \hat{R}+N_{A}Tr^{\left(2\right)}(\Gamma_{A}^{\left(4\right)}){\langle \cdot \rangle }_{2}{\hat{R}}^{2}+Tr^{\left(2\right)}\left(\Gamma_{A}^{\left(2\right)}\right)\Gamma_{B}^{\left(2\right)}{\langle \cdot \rangle }_{2}{\hat{R}}^{2}\\ & -2\hat{R}\cdot \Gamma_{A}^{\left(1\right)}\cdot \Gamma_{B}^{\left(3\right)}\cdot \hat{R} \end{array}\;$$

(A9) $$\begin{array}{ll} \Theta^{\left(44\right)} & =N_{B}{\hat{R}}^{4}{\langle \cdot \rangle }_{4}\Gamma_{A}^{\left(4\right)}-4{\hat{R}}^{3}{\langle \cdot \rangle }_{3}\Gamma_{A}^{\left(3\right)}\Gamma_{B}^{\left(1\right)}\cdot \hat{R}+6{\hat{R}}^{2}{\langle \cdot \rangle }_{2}\Gamma_{A}^{\left(2\right)}\Gamma_{B}^{\left(2\right)}{\langle \cdot \rangle }_{2}{\hat{R}}^{2}-4\hat{R}\cdot \Gamma_{A}^{\left(1\right)}\Gamma_{B}^{\left(3\right)}{\langle \cdot \rangle }_{3}{\hat{R}}^{3}\\ & -N_{A}\Gamma_{B}^{\left(3\right)}{\hat{R}}^{3}{\langle \cdot \rangle }_{3}+N_{A}\Gamma_{B}^{\left(4\right)}{\langle \cdot \rangle }_{4}{\hat{R}}^{4} \end{array}\;$$
